# Phylogenetic Analysis of Foot-and-Mouth Disease Virus from Cattle in Nigeria, 2017–2020

**DOI:** 10.3390/v18080848

**Published:** 2026-08-02

**Authors:** Aliyu Sada, Jibril Adamu, Haruna M. Kazeem, Junaidu Kabir, Hussaini G. Ularamu, Yiltawe Wungak, Kabiru H. Ahmad, Bala N. Umar, Bridget Fomenky, Kate Hole, Sean Yeo, Shawn Babiuk, Oliver Lung, Charles Nfon

**Affiliations:** 1 National Veterinary Research Institute, Vom 930010, Nigeria; aliyuvet@gmail.com (A.S.); ularamuhussaini@yahoo.co.uk (H.G.U.); yiltex2@gmail.com (Y.W.); 2 Department of Veterinary Microbiology, Ahmadu Bello University, Zaria 810211, Nigeria; jiboadm@yahoo.com (J.A.); harunakazeem60@gmail.com (H.M.K.); kabirbka@gmail.com (K.H.A.); umarningi5@gmail.com (B.N.U.); 3 Department of Veterinary Public Health and Preventive Medicine, Ahmadu Bello University, Zaria 810211, Nigeria; junabir@gmail.com; 4 National Centre for Foreign Animal Disease, Canadian Food Inspection Agency, 1015 Arlington Street, Winnipeg, MB R3E 3M4, Canada; bfomenky@yahoo.com (B.F.); kate.hole@inspection.gc.ca (K.H.); sean.yeo@inspection.gc.ca (S.Y.); oliver.lung@inspection.gc.ca (O.L.)

**Keywords:** foot-and-mouth disease, cattle, Nigeria, serotyping, phylogenetic analysis

## Abstract

The aim of this study was to detect and characterize foot-and-mouth disease virus (FMDV) serotypes circulating in cattle from Nigeria and to determine the genetic relationship with FMDV from previous studies in Nigeria. A cross-sectional study was undertaken between 2017 and 2020, during which a total of 234 epithelial tissue samples were collected from reported outbreaks in nine states across Nigeria over a four-year period. Serotypes O/EA-3 and SAT2/VII were identified in samples from 2017 and 2018, serotypes A/Africa/G-IV and SAT2/VII in 2019, and all three serotypes including O/EA-3 and O/WA were identified in samples from 2020. This study identified the presence of three FMDV serotypes (O/EA-3 and O/WA, A/Africa/G-IV and SAT2/VII) circulating in Nigeria. The three serotypes detected in this study can be recommended for production of the appropriate vaccines to control FMDV in Nigeria.

## 1. Introduction

Foot-and-mouth disease (FMD) is a transboundary animal disease that negatively impacts trade and production in livestock and livestock products in Nigeria [1]. FMD is caused by FMD virus (FMDV) of the family *Picornaviridae* and the genus *Aphthovirus*. Seven serotypes of this virus have been identified worldwide which include A, O, C, South African Territories (SAT) 1, SAT 2, SAT 3, and Asia 1 [2]. Serotype C has not been reported since the 2004 outbreaks in Brazil and Kenya and is most likely extinct [2]. For epidemiology, FMDV is characterized into seven pools which contain an unequal distribution of different FDMV serotypes [3]. The African continent contains pool 4, 5, and 6. Pool 4 (East Africa) has serotypes O, A, SAT1, and SAT2 present. Pool 5 (Central and West Africa) has serotypes O, A, SAT1, and SAT2. Pool 6 (Southern Africa) has serotypes SAT1, SAT2 and SAT3. In livestock, FMDV pathogenesis includes an acute symptomatic phase characterized by pyrexia and lesions (vesicles) on the mucus membranes of the tongue, gum, teat, and foot [4]. In some individual cattle, the virus may persist in the pharyngeal region and potentially serve as a source of persistent infection [4].

The first official report of FMD in Nigeria was in 1924 as sporadic outbreaks, with serotype O identified as the causative agent [5]. Subsequently, serotypes A, SAT 1, and SAT 2 were later identified to cause outbreaks across the country [1,6,7,8]. Trade and pastoral cattle entering Nigeria from neighbouring countries through unregulated land borders have been implicated in the spread of FMD in the country [9]. This, coupled with a poor national disease reporting system and poor disease surveillance has contributed to the endemicity and economic consequences of the disease in the country [7,9,10].

Vaccination is an important tool in the control of FMD in Nigeria and is practiced in some commercial dairy farms, although vaccine coverage remains low with small holder farmers. Stamping out of FMDV is not practiced in Nigeria. To be effective, vaccination should target the serotypes and strains/topotypes circulating within Nigeria and the West Africa region, and vaccine coverage should be increased. Molecular characterization of circulating FMDV will allow for the most appropriate vaccines to be used, leading to improved control of the disease [11]. The overall purpose of this study was to sequence the circulating FMDV in Nigeria and to compare the sequences using phylogenetic analysis.

## 2. Materials and Methods

### 2.1. Study Population and Sample Collection

A cross-sectional study with purposive sampling was performed using sedentary and pastoral cattle herds in Nigeria. Study locations were identified based on reports of FMD-suspected animals showing clinical signs presenting as vesicular lesions on the feet, in and around the oral cavity, and on the mammary gland by the Area Veterinary officers. Between 2017 and 2018, 28 samples were collected from 3 states (Oyo, Plateau and Bauchi). In 2019, 28 samples were collected from 5 states (Bauchi, Plateau, Kebbi, Taraba, Lagos). In 2020, 178 samples were collected from 6 states (Bauchi, Plateau, Katsina, Kano, Kaduna, Adamwa). Samples were collected from each suspected outbreak with different cattle displaying clinical disease.

Epithelial tissue samples from un-ruptured and freshly ruptured vesicles were collected. The tissue samples were then stored in labelled sample bottles containing 5X-PSGA transport medium (penicillin, streptomycin, gentamycin and amphotericin-B) diluted in a 1:1 ratio with glycerol. The specimens were transported on gel packs to the FMD laboratory, National Veterinary Research Institute (NVRI), Vom, Nigeria and stored at −80 °C for further processing and/or shipment to the FMD reference laboratory, National Centre for Foreign Animal Disease, Winnipeg, Canada.

### 2.2. Sample Preparation

Epithelial tissues were weighed to create 10% weight/volume tissue suspensions in Dulbecco’s Phosphate-Buffered Saline (D-PBS). Tissues in D-PBS were homogenized using CKMix lysing vials (P000918-LYSK1-A.0, Bertin Technologies, Montigny-le-Bretonneux, France) with a precellys tissue homogenizer (Bertin Technologies) for two rounds at 5000 rpm for 3 × 10 s with a 10 s pause in between. These suspensions were then clarified by centrifugation (2000× *g* for 20 min at 4 °C) prior to RNA extraction. Tissue suspensions were stored at −70 °C until needed.

### 2.3. RNA Extraction and Real-Time Reverse Transciptase-PCR (RT-qPCR)

RNA extraction was performed using the Applied Biosystems MagMax-96 Viral RNA Isolation Kit (AMB18365, ThermoFisher Scientific, Burlington, ON, Canada), following the manufacturer’s protocol. RNA was extracted using a KingFisher APEX (ThermoFisher Scientific) and then assessed for FMDV positivity by RT-qPCR as described previously [12]. Briefly, template RNA (5 μL) was added to a 20 μL mastermix comprising TaqMan^®^ Fast Virus 1-Step master mix (ThermoFisher Scientific), 0.5 μM each of the forward (5′-ACTGGGTTTTAYAAACCTGTGATG-3′) and reverse primers (5′- TCAACTTCTCCTGKATGGTCCCA-3′)**,** and 0.2 μM probe (5′-ATCCTCTCCTTTGCACGC-3′). RT-qPCR was performed on a QuantStudio™ 7 Pro (A43162, ThermoFisher Scientific) machine using the cycling conditions: 50 °C for 5 min, 95 °C for 20 s, followed by 40 cycles of 95 °C for 15 s and 60 °C for 45 s. A cut-off quantification cycle (Cq) value of ≤35.99 was considered positive for the presence of FMDV genome in the sample as this is the cut-off used for diagnostic submission to rule out FDMV [12].

### 2.4. Virus Isolation and Double Antibody Sandwich Enzyme-Linked Immunosorbent Assay (DAS-ELISA)

Virus isolation was performed on a select subset of samples that were tested positive by RT-qPCR to obtain stock virus for further studies. Briefly, monolayers of a porcine kidney cell line expressing αvβ6 integrin (LFBKαvβ6) [13] were inoculated, and the plates were incubated for 2–3 days at 37 °C with daily monitoring for cytopathic effect (CPE). Samples which were negative for virus isolation on the first passage underwent a second cell culture passage [14]. All virus isolation-positive samples were confirmed to be FMDV by RT-qPCR, with the serotype confirmed by DAS-ELISA. The classical DAS-ELISA for FMDV was performed as described previously [15]. An OD450 value of 0.1 was set as a cut-off for all FMDV DAS-ELISAs. When the test sample OD450 was below 0.1, it indicated a negative test; an OD450 value of ≥0.1 represented a positive test [16].

### 2.5. Nanopore (P1) and Whole Genome Sequencing (WGS)

P1 sequences were obtained using nanopore sequencing as previously described with modifications [17]. Briefly, cDNA template was produced from extracted RNA using SuperScript™ IV First-Strand Synthesis System (18091050, ThermoFisher Scientific) and a universal FMDV poly (A) primer. FMDV P1 PCR amplicons were generated from the cDNA template with the Platinum™ SuperFi II PCR Master Mix (12368010, ThermoFisher Scientific) and pan-serotype FMDV primers targeting the P1 region [18]. Amplicons were confirmed with gel electrophoresis first and followed by two cleanup steps with AMPure XP magnetic beads (A63880, Beckman Coulter, Mississauga, ON, Canada). Cleaned amplicons were barcoded with the Native Barcoding Kit (SQK-NBD114, ONT, Oxford, UK) using a modified protocol and then sequenced with Flongle or MinION Flow Cells (FLO-FLG114 or FLO-MIN114, ONT) on a MinION Mk1D (ONT) [17].

Raw sequencing data was processed as done previously [17]. Briefly, reads were mapped using Minimap2 (v2.28) [19] against a custom database of reference FMDV sequences. Mapping statistics and consensus sequences were generated using Samtools (v1.21) [20].

Near full-genome sequences of FMDV were also obtained using next-generation sequencing (NGS). Total RNA was extracted from the samples as above. DNase treatment and RNA purification were performed prior to first-strand cDNA synthesis. Illumina Nextera XT sequencing libraries were prepared according to the manufacturer’s instructions and then sequenced on a MiSeq instrument using a V3 cycling kit (Illumina, Saffron Walden, UK). Raw sequencing data were processed with the nf-v-illuminaNextflow in accordance with Di Tommaso et al.’s [21] workflow (https://github.com/CFIA-NCFAD/nf-villumina accessed on 12 June 2026) for quality control. Taxonomic classification and de novo assembly was performed with Unicycler v0.4.7 in conservative mode to find an optimal SPAdes assembly for each set of paired-end Illumina reads [22]. Contigs were identified as FMDV using nucleotide BLAST search against the NCBI nucleotide database [23]. The gene segments, including VP1, were identified with Annotate in Geneious v9.1.8 using a publicly available annotated FMDV genome, and the VP1 sequences were used for phylogenetic analysis.

Alignments and phylogenetic trees were created in MEGA12 [24] using the VP1 region only. All VP1 sequences were aligned by serotype using MUSCLE (default settings). The best-fit model for the data was determined after alignment and phylogenetic trees were constructed from the chosen model using the Maximum Likelihood method with 1000 bootstrap replicates.

## 3. Results

### 3.1. RT-qPCR and DAS-ELISA

From the 28 samples collected between 2017 and 2018 from three states (Oyo, Plateau and Bauchi), 17 samples were positive by RT-qPCR, with all samples from Oyo State being negative. Of the 17 samples positive by RT-qPCR, 15 were positive on the DAS ELISA with SAT2 and O serotypes identified, and isolates were obtained from 15 samples.

From the 28 samples collected in 2019 from five states (Bauchi, Plateau, Kebbi, Taraba, Lagos), 25 samples were positive by RT-qPCR, and 15 samples were positive by DAS-ELISA which were also positive by RT-qPCR. Only five isolates were obtained, all of which were positive with prior testing by RT-qPCR, with four samples previously identified as positive using prior testing with DAS-ELISA.

From the 178 samples collected in 2020 from six states (Bauchi, Plateau, Katsina, Kano, Kaduna, Adamwa), 169 samples were positive by RT-qPCR, with 18 samples being weak positives with Ct values ≤ 30, and nine samples were negative by RT-qPCR.

Of the 234 samples in total ranging in dates from 2017 to 2020, of which the majority were from 2020, 23 were negative by RT-qPCR either due to poor sampling, sampling an animal that was FMDV negative, or samples that were poorly stored and likely degraded during transport. In general, most samples contained high levels of genome with Cq values < 25.

### 3.2. Sequencing and Phylogenetics

**Figure 1:**
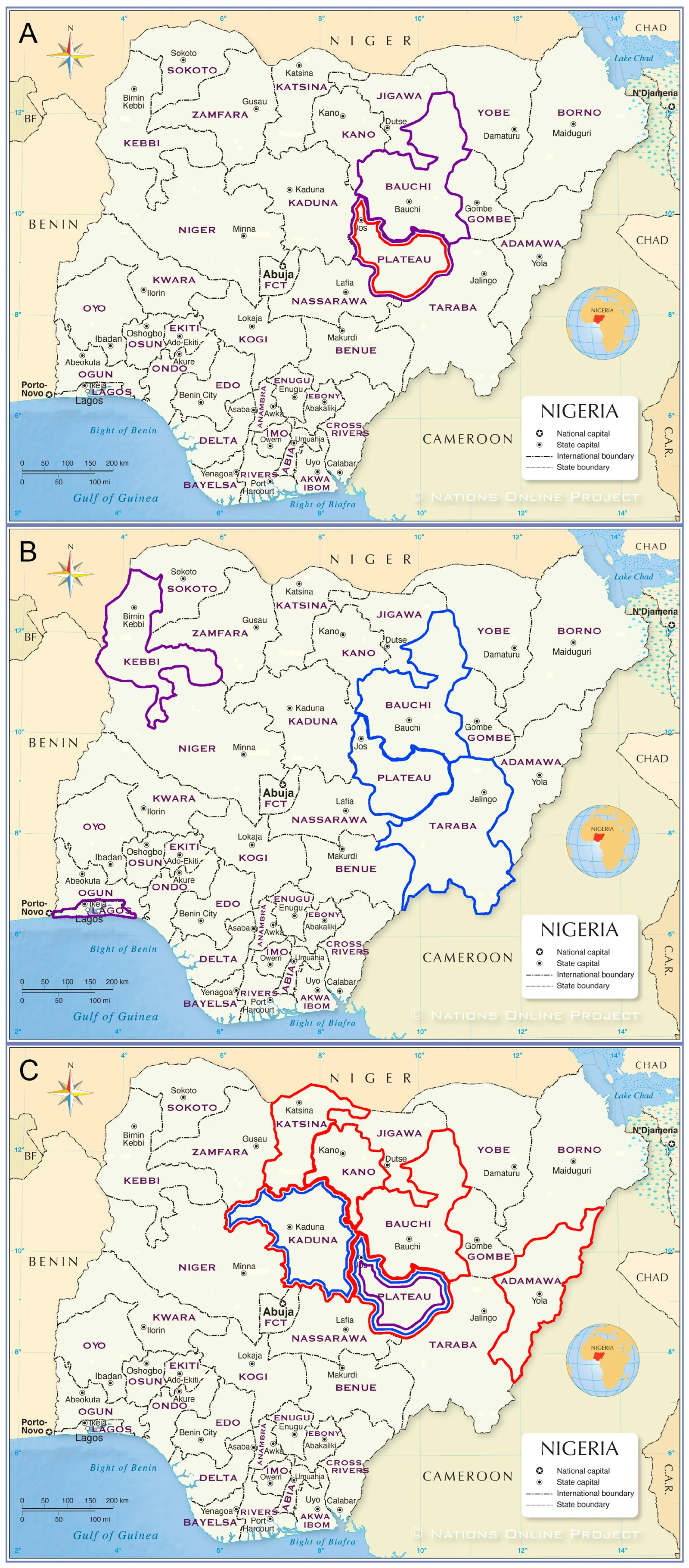
Geographical distribution of FMDV in Nigeria from 2017 to 2020. (**A**). FMDV O and SAT2 serotype distribution in 2017 and 2018. (**B**). FMDV A and SAT2 serotype distribution in 2019. (**C**). FMDV O, A and SAT2 serotype distribution in 2020. FMDV O serotype regions are outlined in red, FMDV A serotype regions are outlined in blue, FMDV SAT2 regions are outlined in purple. Original map source: Nations Online Project (Administrative Map of Nigeria—Nations Online Project, https://www.nationsonline.org/oneworld/map/nigeria-administrative-map.htm accessed on 12 June 2026).

Not all 211 RT-qPCR positive samples were chosen for sequencing; all isolates were sequenced. Samples with high Cq values are difficult to obtain good sequencing data from, and, in some instances, multiple samples were taken from the same area on the same day. For these reasons, not all of the samples were sequenced. Of the 211 positives samples, 108 were sequenced either using Nanopore (P1 region), Illumina (WGS), or both. Sequencing results show that in 2017 and 2018, both O/EA-3 and SAT2/VII serotypes/topotypes were present. In 2019, A/Africa/G-IV and SAT2/VII were present. In 2020, all three FMDV serotypes/topotypes were observed with the addition of FMDV O/WA (Table 1, Figure 1). The geographical distribution shows that multiple serotypes are present per state within Nigeria. From the 116 sequences obtained between 2017 and 2020, 16 sequences were serotype A, 34 sequences were serotype SAT2 and 66 sequences were serotype O.

**Figure 2:**
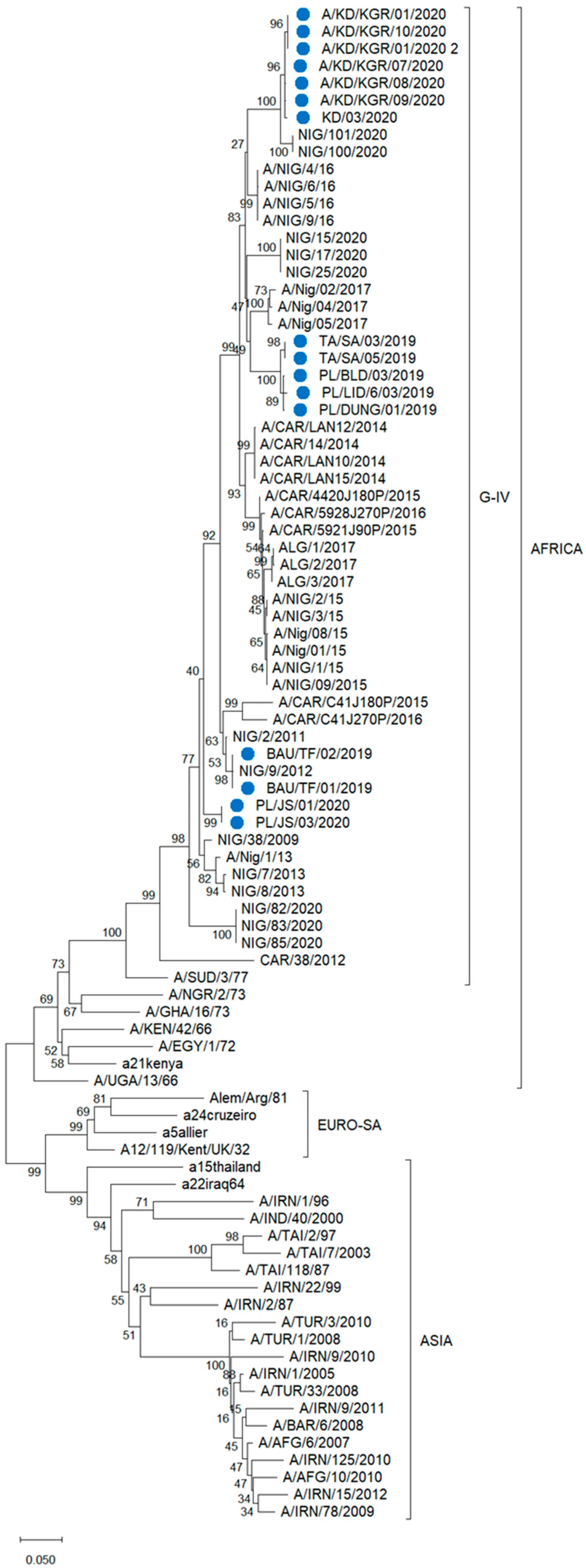
Phylogenetic analysis of FMDV serotype A sequences from 2017 to 2020 detected in cattle from Nigeria. The evolutionary tree for VP 1 nucleotide sequences was constructed using the Maximum Likelihood method and computed using the General Time Reversible model with discrete Gamma distribution and Invariable sites (GTR+G+I) model with MEGA12. The robustness of the phylogenetic tree was assessed with 1000 bootstrap replicates. The blue circles are sequences generated from the samples from this paper.

**Figure 3:**
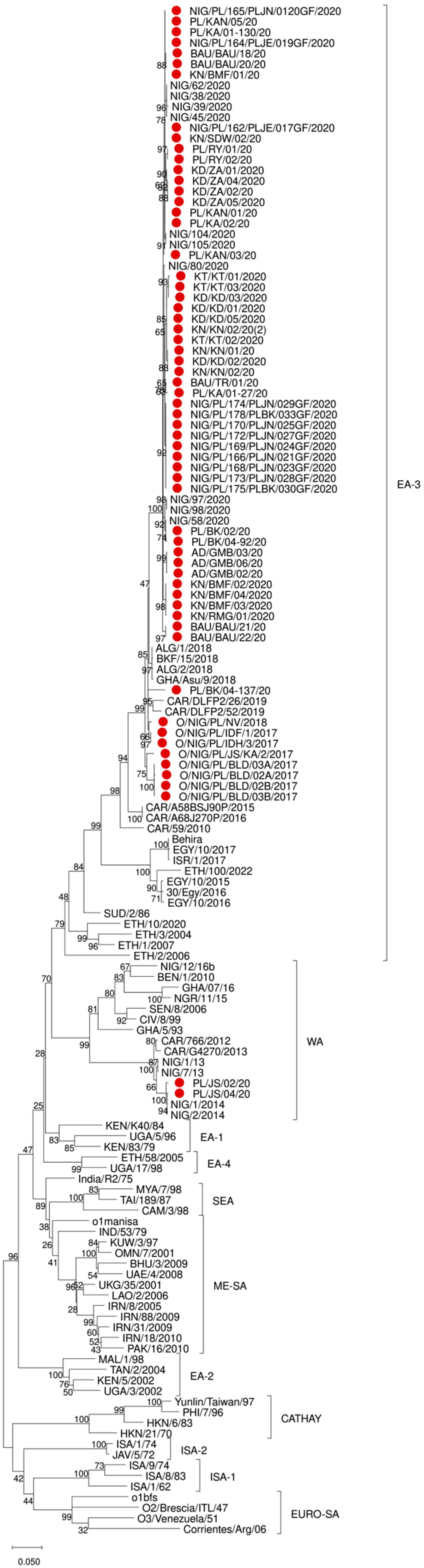
Phylogenetic Analysis of FMDV Serotype O sequences from 2017 to 2020 detected in cattle from Nigeria. The evolutionary tree for VP 1 nucleotide sequences was constructed using the Maximum Likelihood method and computed using the GTR+G+I model with MEGA12. The robustness of the phylogenetic tree was assessed with 1000 bootstrap replicates. The red circles are sequences generated from the samples from this paper.

**Figure 4:**
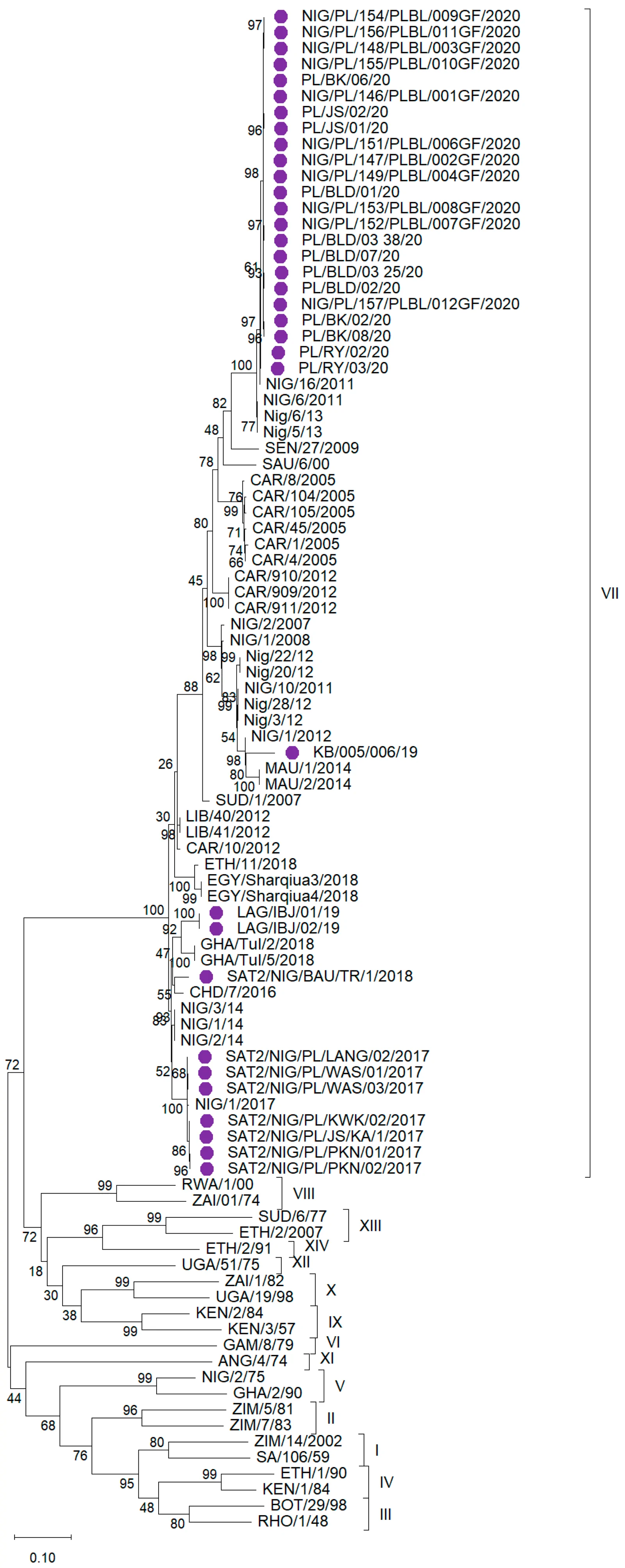
Phylogenetic analysis of FMDV SAT2 sequences from 2017 to 2020 detected in cattle from Nigeria. The evolutionary tree for VP 1 nucleotide sequences was constructed using the Maximum Likelihood method and computed using the Hasegawa–Kishino–Yano (HKY+G+I) model with MEGA12. The robustness of the phylogenetic tree was assessed with 1000 bootstrap replicates. The purple circles are sequences generated from the samples from this paper.

Serotype-specific VP1 sequence data was aligned using MEGA12. The best-fit model was determined for each serotype after alignment, and phylogenetic trees were constructed using the best-fit model with 1000 bootstrap replicates (Figure 2, Figure 3 and Figure 4).

The VP1 sequence data and corresponding phylogenetic analysis of the FMDV serotype A sequences revealed that they belonged to the A/AFRICA/G-IV topotype (Figure 2). Sequences obtained in 2019 formed two different clusters. The TA/SA/03/2019 cluster was most closely related to A/Nig/04/2017, and the BAU/TF/01/2019 cluster was most closely related to NIG/9/2012. In 2020, two additional clusters were identified, with A/KD/KGR/01/2020 clustered sequences most closely related to NIG/100/2020 and the PL/JS/01/2020 clustered sequences that were more distinct with more amino acid differences between different FMDV A viruses identified in Nigeria including NIG/38/2009. The amino acid sequence alignment of the sequences is in Figure S1. The BAU/TF/01/2019 clustered sequences and PL/JS/01/2020 clustered sequences are distinct with several amino acid differences between them in the VP1 region.

The majority of FMDV O serotypes in this study were FMDV/O/EA-3. Samples collected in 2017 and 2018 were clustered together and were most closely related to CAR/DLFP2/26/2019. Samples from 2020 had six clusters that were very closely related with differences ranging between one and two animo acids between the VP1 sequences (Figure S2). These sequences were closely related to sequences for Nigeria (NIG/62/2020, NIG/80/2020, NIG/98/2020 and related sequences). In addition, two FMDV O samples taken from the Plateau state in 2020 were FMDV/O/WA which is most closely related to NIG/1/2014 (Figure 3 and Figure S2).

All FMDV SAT2 sequences were found to fall into the FMDV/SAT2/VII lineage, with the sequences clustering together by the year in which they were collected (Figure 4). The SAT2 sequences obtained from the 2017 cluster SAT2/NIG/PL/KWK/02/2017 and similar sequences were most closely related to NIG/1/17. The SAT2 sequence from the 2018 cluster SAT2/NIG/BAU/TR/1/2018 was most closely related to CHD/7/2016. The SAT2 sequences from 2019 were distinct with SAT2 detected in Kebbi KB/005/006/19 being closest to MAU/2/2014, and Lagos LAG/IBJ/01/19 was most similar to GHA/Tul/2/2018. Kebbi and Lagos locations are geographically distant from the 2017–18 and 2020 positive areas bordering Benin and Niger. SAT2 sequences obtained in 2020 clustered together with small amino acid differences found between the VP1 sequences (Figure S3) and were closely related to NIG/16/2011.

## 4. Discussion

Understanding the specific FMDV circulating in a country is important for the control of the disease by utilizing the most appropriate FMDV vaccines to protect against the current circulating serotypes. Sequence information is particularly important to study FMDV movement in the region. This study focused on sequencing samples from outbreaks of FMDV-suspected cases in Nigeria. Samples were collected from reported outbreaks throughout the country between 2017 and 2020. However, this likely does not reflect the total outbreaks in the country due to the under-reporting of the disease [7]. Because FMD reporting may be incomplete in Nigeria, the results reflect sampled outbreaks rather than national prevalence or true national distribution. In addition, poor sample quality caused from inadequate sampling methods or degradation could impact the study.

Three FMDV serotypes consisting of O/EA-3 (64/116), O/WA (2/116), A/AFRICA/G-IV (16/116) and SAT2/VII (34/116) were identified in Nigeria. Serotype O was the more predominant FMDV serotype primarily of O/EA origin (64/116), with the O/WA topotype (2/116) also being identified in 2020. This agrees with previous studies in Nigeria [5,6,7,8]. Serotype O has been identified as the predominant FMDV serotype in other African countries such as Egypt [25], Sudan [26], Cameroon [27], and Algeria [28,29]. The genetic relatedness between the serotype O/EA-3 isolates that caused outbreaks in Kano, Kaduna, and Katsina states, along with the isolates that circulated in Ghana in 2018 (LC456873), Cameroon in 2012 (KU720578), Libya in 2013 (MG983695), Sudan in 1989 (GU566038) and UAE 2 in 2014 (MG983736), may be accounted for by a transboundary transmission of these viruses across East and West African sub-regions. Livestock movements between Sudan and Central Africa to West Africa [30], population displacement resulting from conflicts caused by the presence of insurgence groups, and attractiveness of the Lake Chad area by the nomadic pastoralists may also contribute to the diffusion of FMD in the region [31]. Serotype A/AFRICA/G-IV has maintained its circulation in the country as previously reported [8,11]. The BAU/TF/01/2019 clustered sequences and PL/JS/01/2020 clustered sequences are distinct with several amino acid differences between them in the VP1 region. There are two possibilities to explain the large differences between these sequences: which are: there is a lack of surveillance in Nigeria, and there are related A/AFRICA/G-IV viruses circulating which would represent the missing viruses from the phylogenetic tress or national cross-border spread occurred through uncontrolled animal movement in regions with a lack of surveillance. SAT2/VII was also detected in Nigeria between 2017 and 2020 in various regions and corroborates the data from a previous study [22]. Since there is animal movement, transboundary trade together with surveillance bias and sampling gaps in Nigeria and the surrounding region make it difficult to trace FMDV outbreaks.

This study agrees with others where serotypes A and SAT2 were identified from recent isolates from Cameroon in Central Africa [32], along with serotypes A/AFRICA/G-IV, O/EA-3, and SAT2/VII identified in Nigeria [33,34]. In this study SAT1/X was not detected in Nigeria as previously observed and described by Ehizibolo [34]. This data also indicates that serotypes O/EA-3, O/WA, A/AFRICA/G-IV and SAT2/VII have remained prevalent within Nigeria over a period of years [5,6,35].

The information described in the paper can be used to inform Nigeria of the circulating FMDV and to demonstrate that FMDV from surrounding regions risks spreading into Nigeria. This has implications for increased border biosecurity and increases awareness of the importance of nomad livestock management. In addition, vaccine matching studies can be performed to ensure that the vaccines selected for use are effective against the current circulating FMDV.

## 5. Conclusions

To our knowledge, this may be the first report on the characterization of FMDV in several states of Nigeria. This report provides important information about FMDV O, A and SAT2/VII serotypes/topotypes circulating in Nigeria during the study period. This information provides insight into the lineages that require vaccine coverage to develop a multivalent vaccine for the control of FMD in Nigeria.

## Figures and Tables

**Table 1:** Distribution of FMDV serotypes in Nigeria from 2017 to 2020.

Year	Sample ID	Region State/Local Government Area	Serotype	Topotype/Lineage	Accession #
2017	PL/IDF/1	Plateau/Jos South	O	EA-3	OM022648
2017	PL/IDF/3	Plateau/Jos South	O	EA-3	OM022649
2017 *	PL/LANG/02	Plateau/Langtang	SAT2	VII	MW715625
2017 *	PL/WAS/01	Plateau/Wase	SAT2	VII	MW715628
2017 *	PL/WAS/03	Plateau/Wase	SAT2	VII	MW715629
2017 *	PL/PKN/01	Plateau/Pankshin	SAT2	VII	MW715626
2017 *	PL/PKN/02	Plateau/Pankshin	SAT2	VII	MW715627
2017	PL/BLD/02A	Plateau/B-Ladi	O	EA-3	OM022644
2017	PL/BLD/03A	Plateau/B-Ladi	O	EA-3	OM022646
2017	PL/BLD/02B	Plateau/B-Ladi	O	EA-3	OM022645
2017	PL/BLD/03B	Plateau/B-Ladi	O	EA-3	OM022647
2017	PL/JS/KA/2/17	Plateau/Jos South	O	EA-3	OM022643
2017 *	PL/JS/KA/1/17	Plateau/Jos South	SAT2	VII	MW715623
2017 *	PL/KWK/02	Plateau/Langtang	SAT2	VII	MW715624
2018	PL/NV/2018	Plateau/Jos South	O	EA-3	OM022642
2018 *	BAU/TR/1	Bauchi/Toro	SAT2	VII	MW715622
2019	TA/SA/03/19	Taraba	A	Africa/G-IV	PZ747309
2019	TA/SA/05/19	Taraba	A	Africa/G-IV	PZ747310
2019	PL/BLD/03/19	Plateau	A	Africa/G-IV	PZ581628
2019	PL/DUNG/1	Plateau	A	Africa/G-IV	PZ581629
2019	PL/LID/6/03/19	Plateau	A	Africa/G-IV	PZ581627
2019	KB/005/006/19	Kebbi	SAT2	VII	PZ747311
2019	BAU/TF/01/19	Bauchi	A	Africa/G-IV	PZ747312
2019	BAU/TF/02/19	Bauchi	A	Africa/G-IV	PZ581631
2019	LAG/IBJ/01/19	Lagos	SAT2	VII	PZ747313
2019	LAG/IBJ/02/19	Lagos	SAT2	VII	PZ747314
2020	KD/03/20	Kaduna	A	Africa/G-IV	PZ581630
2020	PL/JS/01/20	Plateau	A	Africa/G-IV	PZ581632
2020	PL/JS/02/20	Plateau	O	WA	PZ747315
2020	PL/JS/03/20	Plateau	A	Africa/G-IV	PZ581633
2020	PL/JS/04/20	Plateau	O	WA	PZ747316
2020	PL/KA/01/20	Plateau	O	EA-3	PZ747324
2020	BAU/TR/01/20	Bauchi/Toro	O	EA-3	PZ747334
2020	KN/KN/01/20	Kano/Kano	O	EA-3	PZ747329
2020	KN/KN/02/20	Kano/Kano	O	EA-3	PZ747330
2020	KT/KT/01/20	Katsina/Katsina	O	EA-3	PQ662955
2020	KT/KT/02/20	Katsina/Katsina	O	EA-3	PQ662956
2020	KT/KT/03/20	Katsina/Katsina	O	EA-3	PQ662957
2020	KD/KD/01/20	Kaduna/Kaduna	O	EA-3	PQ662938
2020	KD/KD/02/20	Kaduna/Kaduna	O	EA-3	PQ662939
2020	KD/KD/03/20	Kaduna/Kaduna	O	EA-3	PQ662940
2020	KD/KD/05/20	Kaduna/Kaduna	O	EA-3	PQ662941
2020	PL/KAN/01/20	Plateau	O	EA-3	PZ747338
2020	PL/KAN/03/20	Plateau	O	EA-3	PZ747339
2020	PL/KAN/05/20	Plateau	O	EA-3	PZ747340
2020	PL/KA/01/20	Plateau	O	EA-3	PZ747344
2020	PL/KA/02/20	Plateau	O	EA-3	PZ747345
2020	PL/RY/01/20	Plateau	O	EA-3	PZ747346
2020	PL/RY/02/20	Plateau	O	EA-3	PZ747347
2020	PLJE 016GF	Plateau/Jos East (JE)	O	EA-3	OQ457183
2020	PLJE 017GF	Plateau/Jos East (JE)	O	EA-3	OQ457184
2020	PLJE 019GF	Plateau/Jos East (JE)	O	EA-3	OQ457185
2020	PLBK 030GF	Plateau/Bokkos (BK)	O	EA-3	OQ457194
2020	PLBK 033GF	Plateau/Bokkos (BK)	O	EA-3	OQ457195
2020	KD/KGR/01/20	Kaduna/Kajuru	A	AFRICA/G-IV	PQ662942
2020	KD/KGR/03/20	Kaduna/Kajuru	A	AFRICA/G-IV	PQ662943
2020	KD/KGR/07/20	Kaduna/Kajuru	A	AFRICA/G-IV	PQ662944
2020	KD/KGR/08/20	Kaduna/Kajuru	A	AFRICA/G-IV	PQ662945
2020	KD/KGR/09/20	Kaduna/Kajuru	A	AFRICA/G-IV	PQ662946
2020	KD/KGR/10/20	Kaduna/Kajuru	A	AFRICA/G-IV	PQ662947
2020	PL/JS/01/20	Plateau	SAT2	VII	PZ747332
2020	PL/JS/02/20	Plateau	SAT2	VII	PZ747333
2020	AD/GMB/02/20	Adamawa	O	EA-3	PZ747341
2020	AD/GMB/03/20	Adamawa	O	EA-3	PZ747342
2020	AD/GMB/06/20	Adamawa	O	EA-3	PZ747343
2020	BAU/BAU/18/20	Bauchi/Bauchi	O	EA-3	PZ747317
2020	BAU/BAU/20/20	Bauchi/Bauchi	O	EA-3	PZ747318
2020	BAU/BAU/21/20	Bauchi/Bauchi	O	EA-3	PZ747319
2020	BAU/BAU/22/20	Bauchi/Bauchi	O	EA-3	PZ747320
2020	PL/BK/04/20	Plateau	O	EA-3	PZ747337
2020	PL/BK/06/20	Plateau	SAT2	VII	PZ747349
2020	KN/BMF/01/20	Kano/Bunkure	O	EA-3	OR351383
2020	KN/BMF/02/20	Kano/Bunkure	O	EA-3	PQ662951
2020	KN/BMF/03/20	Kano/Bunkure	O	EA-3	PQ662952
2020	KN/BMF/04/20	Kano/Bunkure	O	EA-3	PQ662953
2020	KN/RMG/01/20	Kano/Rimin Gado	O	EA-3	PQ662954
2020	PL/BLD/03/20	Plateau/B-Ladi	SAT2	VII	PZ747325
2020	PL/BLD/07/20	Plateau/B-Ladi	SAT2	VII	PZ747326
2020	PLBL 001GF	Plateau/B-Ladi	SAT2	VII	OQ401864
2020	PLBL 002GF	Plateau/B-Ladi	SAT2	VII	OQ401865
2020	PLBL 003GF	Plateau/B-Ladi	SAT2	VII	OQ401866
2020	PLBL 004GF	Plateau/B-Ladi	SAT2	VII	OQ401867
2020	PLBL 006GF	Plateau/B-Ladi	SAT2	VII	OQ401868
2020	PLBL 007GF	Plateau/B-Ladi	SAT2	VII	OQ401869
2020	PLBL 008GF	Plateau/B-Ladi	SAT2	VII	OQ401870
2020	PLBL 009GF	Plateau/B-Ladi	SAT2	VII	OQ401871
2020	PLBL 010GF	Plateau/B-Ladi	SAT2	VII	OQ401872
2020	PLBL 011GF	Plateau/B-Ladi	SAT2	VII	OQ401873
2020	PLBL 012GF	Plateau/B-Ladi	SAT2	VII	OQ401874
2020	PL/BK/04/20	Plateau	O	EA-3	PZ747348
2020	PLJN 020GF	Plateau/Jos North (JN)	O	EA-3	OQ457186
2020	PLJN 021GF	Plateau/Jos North (JN)	O	EA-3	OQ457187
2020	PLJN 023GF	Plateau/Jos North (JN)	O	EA-3	OQ457188
2020	PLJN 024GF	Plateau/Jos North (JN)	O	EA-3	OQ457189
2020	PLJN 025GF	Plateau/Jos North (JN)	O	EA-3	OQ457190
2020	PLJN 027GF	Plateau/Jos North (JN)	O	EA-3	OQ457191
2020	PLJN 028GF	Plateau/Jos North (JN)	O	EA-3	OQ457192
2020	PLJN 029GF	Plateau/Jos North (JN)	O	EA-3	OQ457193
2020	PL/BK/02/20	Plateau	O	EA-3	PZ581634
2020	PL/RY/02/20	Plateau	SAT2	VII	PZ747335
2020	PL/RY/03/20	Plateau	SAT2	VII	PZ747336
2020	PL/BK/02/20	Plateau	SAT2	VII	PZ747327
2020	PL/BK/08/20	Plateau	SAT2	VII	PZ747328
2020	KD/ZA/01/20	Kaduna/Zaria	O	EA-3	PQ662948
2020	KD/ZA/02/20	Kaduna/Zaria	O	EA-3	OR351384
2020	KD/ZA/04/20	Kaduna/Zaria	O	EA-3	PQ662949
2020	KD/ZA/05/20	Kaduna/Zaria	O	EA-3	PQ662950
2020	KN/SDW/02/20	Kano	O	EA-3	PZ747331
2020	PL/BLD/01/20	Plateau/B-Ladi	SAT2	VII	PZ747321
2020	PL/BLD/02/20	Plateau/B-Ladi	SAT2	VII	PZ747322
2020	PL/BLD/03/20	Plateau/B-Ladi	SAT2	VII	PZ747323

* 2017 SAT2 sequences were previously published by Fomenky et al. [22]. Colours signify the serotype, with peach representing serotype O, blue serotype A and purple serotype SAT2.

## Data Availability

Part of the data from this study is deposited on the Genbank. Data from this study can be obtained from the corresponding authors.

## Supplementary Materials

The following supporting information can be downloaded at: https://www.mdpi.com/article/10.3390/v18080848/s1. Figure S1. Amino acid sequence alignment to the A/AFRICA/GIV prototype A/SUD/3/77, Figure S2. Amino acid sequence alignment to the O/EA-3 prototype ETH/1/2007, Figure S3. Amino acid sequence alignment to the SAT2/VII prototype CAR/1/2005.
