# Supplementary material for: Phylogenetic Analysis of Foot-and-Mouth Disease Virus from Cattle in Nigeria, 2017–2020
Source: Viruses. 2026 Aug 2;18(8):848. doi: 10.3390/v18080848 (PMC13517661; doi:10.3390/v18080848)
Supplement: Supplementary file 1 [file viruses-18-00848-s001.zip › viruses-4410183-supplementary.pdf]

```
 A/SUD/3/77            TATGESADPVTTTVENYGGETQVQRRHHTDVGFIMDRFVKVKSLGPTHVIDLMQTHQHGLVGALLRAATYYFSDLEIVVKHQGNLTWVPNGAPEAALDNTSNPTAYHKAPFTRLALPYTAPHRVLATVYNGTSKYSTGASARRGDLGPLAARVAAQLPASFNYGALRAENIHELLVRMKRAELYCPRPLLAVEVTSADRHKQKIIAPA  208
*A/KD/KGR/01/2020      ......................I........S........IN.PT....V................S.........V..R.................A.M.....................................S..P.................S..........T....................P...SA............  208
*A/KD/KGR/01/2020_2    ......................I........S........IN.PT....V................S.........V..R.................A.M.....................................S..P.................S..........T....................P...SA............  208
*A/KD/KGR/10/2020      ......................I........S........IN.PT....V................S.........V..R.................A.M.....................................S..P.................S..........T....................P...SA............  208
*A/KD/KGR/08/2020      ......................I........S........IN.PT....V................S.........V..R.................A.M.....................................S..P.................S..........T....................P...SA............  208
*A/KD/KGR/09/2020      ......................I........S........IN.PT....V................S.........V..R.................A.M.....................................S..P.................S..........T....................P...SA............  208
*A/KD/KGR/07/2020      ......................I........S........IN.PT....V................S.........V..R.................A.M.....................................S..P.................S..........T....................P...SA............  208
*KD/03/2020            ......................I........S........IS.PT....V................S.........V..R.................A.M.....................................S..P.................S..........T....................P...SA............  208
*PL/LID/6/03/2019      ......................I.................IN.ST.....................S.........V....................A........Y..........................R......P......S......T...S..........T.Q..................P...STT...........  208
*PL/BLD/03/2019        ......................I.................IN.ST.....................S.........V....................A........Y..........................R......P......S......T...S..........T.Q..................P...STT...........  208
*PL/DUNG/01/2019       ......................I.................IN.ST.....................S.........V....................A.A......Y..........................R......P......S......T...S..........T.Q..................P...STT...........  208
*TA/SA/03/2019         ......................I.................IN.ST.....................S.........V....................A........Y..........................R....V.P......S......T...S..........T.Q..................P...STT...........  208
*TA/SA/05/2019         ......................I.................IN.ST.....................S.........V....................A........Y..........................R....V.P......S......T...S..........T.Q..................P...STT...........  208
*BAU/TF/01/2019        .........................................N.PT.....................S.........V....................A.M........................................S......H......T...S..........T.Q...I..............P...SA............  208
*BAU/TF/02/2019        .........................................N.PT.....................S.........V....................A.M........................................S......H......T...S..........T.Q...I..............P...SA............  208
*PL/JS/01/2020         ...............................S........IN.PT.....................S.........V..R..............S..A..................................N.......S.....A...........S..........T.Q...I..............P...SA............  208
*PL/JS/03/2020         ...............................S........IN.PT.....................S.........V..R..............S..A..................................N.......S.....A...........S..........T.Q...I..............P...SA............  208
⁑NIG/100/2020          ......................I........S........IN.PT....V................S.........V..R.................A.M.....................................S..P......S..........S..........T....................P...SAT..Y........  208
⁑NIG/25/2020           ......................I.................IN.PT.....................S.........V....................G.V........................................P......A......T...S..........T.Q...I......F.......P...STG............  208
⁑NIG/83/2020           ........................................IN..AS..........E.........S.........V......................M................................N.....T.S......H..........S..........A.Q..................P...SAK............  208
⁑A/Nig/04/2017         ........................................IN.ST.....................S.........V.................T..A.M........................................P......S.....TT...S..........T.Q..................P...SG............  208
⁑A/NIG/6/16            ......................T.................IS.PT.....................S.........V..R.................A.M........................................PT.......V....T...S..........T.QD..I..............P...SA............  208
⁑A/NIG/3/15            ..M...................T.................IGGST.....................S.........V....................A.M.......................................TST.....S......T...S..........T.Q...I..............P...SA............  208
⁑NIG/8/2013            .................D.....R.................N.PT.....................S.........V....................A..........................................P......A..........S..........T.Q..................P...SA............  208
⁑NIG/9/2012            .........................................N.PT.....................S.........V....................A.M........................................S......H......T...S..........T.Q...I..............P...SA............  208
⁑NIG/2/2011            .........................................N.PT.....................S.........V....................A.M........................................S.............T...S..........T.Q...I..............P...SA............  208
⁑NIG/38/2009           .........................................N.ST.....................S.........V....................A.M................................N.......P......A......T...S..........T.Q...I..............P...SA............  208
⁑ALG/3/2017            ..M...................T.................IG.PT.....................S.........V....................A.M.......................................TSA.....S......T...P..........T.Q...I..............P...SA............  208
⁑A/CAR/LAN15/2014      ......................I......E..........IN.PT.....................S.........V....................A.M.......................................TP......S......T...S..........T.Q...I..............P...SA............  208
⁑A/CAR/5928J270P/2016  ......................A.................IG.ST.....................S.........V....................A.M.......................................TST.....S......T...S..........T.Q...I..............P...SA............  208
⁑A/CAR/C41J180P/2015   ........................................IN.PA.....................S.........V................V.....M........E.......................N......PS......A......T...S..........A.Q...I..............P...SV......Q.....  208
⁑CAR/38/2012           .........................................P.ST.I...................S.........V.A..................N......V...............................ES..P......A..........S..........A.Q..................PT..E.............  208
∮A/NGR/2/73            .........................................NRPS.I..................................E...............H..................................N...A.VQT......S..........T.........DT........................STQ...........  208
∮A/GHA/16/73           .T..............L.......................INNSS.E................................R.D...............Q........................................G.T...........................DT.R......................S.............  208
∮A/KEN/42/66           ......................I.................LN.SS...............................V..R.E.T.............Q.M......................................VTS......S...KA...............TT.....................T..S.............  208
∮A/EGY/1/72            ..A..........................S....L......NNPN..................M...............R.E............S..L..................................T..A.ES.G.........................K.TT........................S.Q...........  208
∮a21kenya              .........................................N.SS.M..................................E...............L..........E............................SV.S...E.......I.............K.T...........................P...........  208
∮A/UGA/13/66           ...................D...R..Q.............IS..S..................................R.D...............Q.M....................................VS.AP......S....................TT........................S.S...........  208
∮a24cruzeiro           ......................I.......I.........IQ..S..................................R.E............S..L........N............................AV.G.G....M.S.....VK..........IK.DA.....................I..S.Q...........  208
∮a5allier              ..V...................T.................IN..S..................................R.D...............S........N.........................N....DG-P....M.S....A.K..........I..VT.....................I..S.Q...........  207
∮a22iraq64             .T........................Q....T........IQN.N..................................R.D...............S..G.....L.............................A.GTG.....................F..IQ.TT........................S.Q...........  208
∮A/IRN/1/2005          .TA...................A...Q.............ISPVS.............A....................R.D...........VE..A..........Q......................V.....TGNG.................S...F..I..TT........................L.Q..........T  208
```

Figure S1. Amino acid sequence alignment to the A/AFRICA/GIV prototype A/SUD/3/77 where * represents samples sequenced in this study, ⁑ are similar sequence data obtained from genbank and ∮ represents serotype A prototypes.

```
 ETH/1/2007                 TTSPGESADPVTATVENYGGETQVQRRQHTDVSFILDRFVKVTP??DQINVLDLMQTPAHTLVGALLRTSTYYFADLEVAVKHEGNLTWVPNGAPESALDNTTNPTAYHKAPLTRLALPYTAPHRVLATVYNGNCKYGE-TPVTNVRGDLQVLAQKVARTLPTSFNYGAIKATRVTELLYRMKRAETYCPRPLLAIHPSEARHKQKIVAPVK  211
*NIG/PL/165/PLJN/0120GF/2020 ...L................Q...........A.......................A............A..........................T.....................................S..TG-A.A.............A..K......................................T.............  211
*PL/KAN/05/20               ...L................Q...........A.......................A............A..........................T.....................................S..TG-A.A.............A..K......................................T.............  211
*PL/KA/01-130/20            ...L................Q...........A.......................A............A..........................T.....................................S..TG-A.A.............A..K......................................T.............  211
*NIG/PL/164/PLJE/019GF/2020 ...L................Q...........A.......................A............A..........................T.....................................S..TG-A.A.............A..K......................................T.............  211
*BAU/BAU/18/20              ...L................Q...........A.......................A............A..........................T.....................................S..TG-A.A.............A..K......................................T.............  211
*BAU/BAU/20/20              ...L................Q...........A.......................A............A..........................T.....................................S..TG-A.A.............A..K......................................T.............  211
*KN/BMF/01/20               ...L................Q...........A.......................A............A..........................T.....................................S..TG-A.A.............A..K......................................T.............  211
*NIG/PL/162/PLJE/017GF/2020 ...L................Q...........A.......................A............A..........................T.....................................S..TG-A.A.............A..K......................................T.............  211
*KN/SDW/02/20               ...L................Q...........A.......................A............A..........................T.....................................S..TG-A.A.............A..K......................................T.............  211
*PL/RY/01/20                ...L................Q...........A.......................A............A..........................T.....................................S..TG-A.A.............A..K......................................T.............  211
*PL/RY/02/20                ...L................Q...........A.......................A............A..........................T.....................................S..TG-A.A.............A..K......................................T.............  211
*KD/ZA/01/2020              ...L................Q...........A.......................A............A..........................T.....................................S..TG-A.A.............A..K......................................T.............  211
*KD/ZA/04/2020              ...L................Q...........A.......................A............A..........................T.....................................S..TG-A.A.............A..K......................................T.............  211
*KD/ZA/02/20                ...L................Q...........A.......................A............A..........................T.....................................S..TG-A.A.............A..K......................................T.............  211
*KD/ZA/05/2020              ...L................Q...........A.......................A............A..........................T.....................................S..TG-A.A.............A..K......................................T.............  211
*PL/KAN/01/20               ...L................Q...........A.......................A............A..........................T.....................................S..TG-A.A.............A..K......................................T.............  211
*PL/KA/02/20                ...L................Q...........A.......................A............A..........................T.....................................S..TG-A.A.............A..K......................................T.............  211
*PL/KAN/03/20               ....................Q...........A.......................A............A..........................T.....................................S..TG-A.A.............A..K......................................T.............  211
*KT/KT/01/2020              ...L................Q...........A.......................A............A..........................T...I.................................S..TG-A.A.............A..K......................................T.............  211
*KT/KT/03/2020              ...L................Q...........A.......................A............A..........................T...I.................................S..TG-A.A.............A..K......................................T.............  211
*KD/KD/03/2020              ...L................Q...........A.......................A............A..........................T...I.................................S..TG-A.A.............A..K......................................T.............  211
*KD/KD/01/2020              ...L....-...........Q...........A.......................A............A..........................T.....................................S..TG-A.A.............A..K......................................T.............  210
*KD/KD/05/2020              ...L................Q...........A.......................A............A..........................T.....................................S..TG-A.A.............A..K......................................T.............  211
*KN/KN/02/20_2              ...L................Q...........A.......................A............A..........................T.....................................S..TG-A.A..........T..A..K......................................T.............  211
*KT/KT/02/2020              ...L................Q...........A.......................A............A..........................T.....................................S..TG-A.A.............A..K......................................T.............  211
*KN/KN/01/20                ...L................Q...........A.......................A............A..........................T.....................................S..TG-A.A..........T..A..K......................................T.............  211
*KD/KD/02/2020              ...L................Q...........A.......................A............A..........................T.....................................S..TG-A.A..........T..A..K......................................T.............  211
*KN/KN/02/20                ...L................Q...........A.......................A............A..........................T.....................................S..TG-A.A.............A..K......................................T.............  211
*BAU/TR/01/20               ....................Q...........A.......................A............A..........................T.....................................S..TG-A.A.............A..K......................................T.............  211
*PL/KA/01-27/20             ....................Q...........A.......................A............A..........................T.....................................S..TG-A...............A..K......................................T.............  211
*NIG/PL/174/PLJN/029GF/2020 ....................Q...........A.......................A............A..........................T.....................................S..TG-A.A.............A..K......................................T.............  211
*NIG/PL/178/PLBK/033GF/2020 ....................Q...........A.......................A............A..........................T.....................................S..TG-A.A.............A..K......................................T.............  211
*NIG/PL/170/PLJN/025GF/2020 ....................Q...........A.......................A............A..........................T.....................................S..TG-A.A.............A..K......................................T.............  211
*NIG/PL/172/PLJN/027GF/2020 ....................Q...........A.......................A............A..........................T.....................................S..TG-A.A.............A..K......................................T.............  211
*NIG/PL/169/PLJN/024GF/2020 ....................Q...........A.......................A............A..........................T.....................................S..TG-A.A.............A..K......................................T.............  211
*NIG/PL/166/PLJN/021GF/2020 ....................Q...........A.......................A............A..........................T.....................................S..TG-A.A.............A..K......................................T.............  211
*NIG/PL/168/PLJN/023GF/2020 ....................Q...........A.......................A............A..........................T.....................................S..TG-A.A.............A..K......................................T.............  211
*NIG/PL/173/PLJN/028GF/2020 ....................Q...........A.......................A............A..........................T.....................................S..TG-A.A.............A..K......................................T.............  211
*NIG/PL/175/PLBK/030GF/2020 ....................Q...........A.......................A............A..........................T.....................................S..TG-A.A.............A..K......................................T.............  211
*PL/BK/02/20                ....................Q...........A.......................A............A..........................T.....................................S..TG-A.A.............A..K......................................T.............  211
*PL/BK/04-92/20             ....................Q...........A.......................A............A..........................T.....................................S..TG-A.A.............A..K......................................T.............  211
*AD/GMB/03/20               ....................Q...........A..................T....A............A..........................T.....................................S..TG-A.A.............A..K......................................T.............  211
*AD/GMB/06/20               ....................Q...........A..................T....A............A..........................T.....................................S..TG-A.A.............A..K......................................T.............  211
*AD/GMB/02/20               ....................Q...........A..................T....A............A..........................T.....................................S..TG-A.A.............A..K......................................T.............  211
*KN/BMF/02/2020             ....................Q...........A.......................A............A..........................T.....................................S..TG-A.A...........R.T..K......................................T.............  211
*KN/BMF/04/2020             ....................Q...........A.......................A............A..........................T.....................................S..TG-A.A...........R.T..K......................................T.............  211
*KN/BMF/03/2020             ....................Q...........A.......................A............A..........................T.....................................S..TG-A.A...........R.T..K......................................T.............  211
*KN/RMG/01/2020             ....................Q...........A.......................A............A..........................T.....................................S..TG-A.A...........R.T..K......................................T.............  211
*BAU/BAU/21/20              ....................Q...........A.......................A............A..........................T.....................................S..TG-A.A.............A..K......................................T.............  211
*BAU/BAU/22/20              ....................Q...........A.......................A............A..........................T.....................................S..TG-A.A.............A..K......................................T.............  211
*PL/BK/04-137/20            ...S........S.......Q...........A.............N.........A............A..........................T.....................................S...R-A...............A.LE...............I......................T.............  211
*O/NIG/PL/NV/2018           ....................Q...........A.......................A............A..........................T.....................................S...G-A...............A..K......................................T.............  211
*O/NIG/PL/IDF/1/2017        ....................Q...........A.......................A............A..........................T.....................................S...G-A...............A..K......................................T.............  211
*O/NIG/PL/IDH/3/2017        ....................Q...........A.......................A............A..........................T.....................................S...G-A...............A..K......................................T.............  211
*O/NIG/PL/JS/KA/2/2017      ...L................Q...........A.......................A............A..........................T.....................................S...G-A.A.............A..K......................................T...........E.  211
*O/NIG/PL/BLD/03A/2017      ....................Q...........A.......................A............A..........................T.....................................S..SG-A...............A..K........................V.............T...........E.  211
*O/NIG/PL/BLD/02A/2017      ....................Q...........A.......................A............A......I...................T.....................................S..SG-A...............A..K........................V.............T...........E.  211
*O/NIG/PL/BLD/02B/2017      ....................Q...........A.......................A............A..........................T.....................................S..SG-A...............A..K........................V.............T...........E.  211
*O/NIG/PL/BLD/03B/2017      ....................Q...........A.......................A............A..........................T.....................................S..SG-A...............A..K........................V.............T...........E.  211
*PL/JS/02/20                ...A...................I......................A.V..I................AA......................................Y.........................S..SGTVAAQ.......T......QA...................................T................  212
*PL/JS/04/20                ...A...................I......................A.V..I................AA......................................Y.........................R..RRPVAAQ.......T......QA...................................T................  212
∮GHA/5/93                   .......................T......................E.........A............A...............D................................................S..SR-VE.PK.........RRA.........F............................V....T.........M.  211
‡NIG/62/2020                ...L................Q...........A.......................A............A..........................T.....................................S..TG-A.A.............A..K......................................T.............  211
‡NIG/80/2020                ....................Q...........A.......................A............A..........................T.....................................S..TG-A.A.............A..K......................................T.............  211
‡NIG/98/2020                ....................Q...........A.......................A............A..........................T.....................................S..TG-A.A.............A..K......................................T.............  211
‡GHA/Asu/9/2018             ....................Q...........A...............V.......A............A..........................T.....................................S...G-A...............A..K......................................T.............  211
‡CAR/DLFP2/26/2019          ....................Q...........A...............F.......A............A..........................T.....................................S...G-A...............A..N....................................................  211
‡ETH/100/2022               ....................V...........A.............S.T.......I.S.........SA..........................T.....................................S...G-ARAI............A................Q.A......................T...........M.  211
‡30/Egy/2016                ....................V...........A.............S.T.......I.T.........SA..........................T.....................................S....-ARAA............A................Q........................T.............  211
‡NIG/1/2014                 ...A...................I......................A.V..I................AA......................................Y.........................S..SG-VAAQ.......T......QA...................................T................  211
‡CAR/766/2012               ...A...................I......................E.V..V................AA................................................................S..SG-VAAQ.......T....A.QA....................................................  211
‡NIG/7/13                   ...A...................I......................E.V..I................AA................................................................S..SG-VAAQ.......T....A.QA....................................................  211
∮UGA/5/96                   ...L..........................................EGT....................A...........V.D.E.........Q.......................................R..V-............V...A.....................................................AR  211
∮ETH/58/2005                ...S..........................................N......................A........................................E...........................T-A..A............A..................I...................V................  211
∮MYA/7/98                   ...T.......................H..............................P..........A...............D..........A.N......................................A.-GSL.............A..P...................................V..D.............  211
∮UKG/35/2001                ...A.................................................................A..........................T..........................................-S...............A.......................................................  211
∮IRN/88/2009                ...T.................................................................A..........................T..........................................-SST.............A..................N......................N.............  211
∮Yunlin/Taiwan/97           ...A...........................IA.........K...E.V.......I............A....S..L.......D..........T.............E.......................SS..D-.STN............AE........F.............................Q..D.....R....A.  211
∮JAV/5/72                   ...A....................R...............................I........G.VAA....S..L..................A.............E..........................TS-VAMA.G.......T..A..P..............LV..........A.........Q...............  211
∮ISA/8/83                   ...A..........................................N.........I...........AA....S..L..V...............A.N.S............................A...E.R..T-HTT.............A..................I.....I............................T.  211
∮o1bfs                      ...A........T..........I...........M..........N...I.....V.S.........A.....S..I.......D..........K....................................E.R.SR-NA.P.L....................................................T.............  211
```

Figure S2. Amino acid sequence alignment to the O/EA-3 prototype ETH/1/2007 where * represents samples sequenced in this study, ‡ are similar sequence data obtained from genbank and ∮ represents serotype O prototypes inc;uding O/WA prototype GHA/5/93.

```
 CAR/1/2005                 TTSAGEGADVVTTDPSTHGGNVQEGRRKHTETAFLLDRSTHVHTNKTSFVVDLMDTKEKALVGAILRASTYYFCDLEIACVGDHTRVFWQPNGAPRATQLGDNPMVFAKGGVTRFAIPFTAPHRLLSTVYNGECSYAKTATPIRGDRAALAAKYARSNHTLPSTFNFGFVTADKPVDVYYRMKRAELYCPRPLLPTYDHAGRDRFDAPIGVERQTL  216
*SAT2/NIG/PL/KWK/02/2017    ........G...............S......V............G.......................................A...........T.....................................D.NR.V.A.............DYT....P................................A....................  216
*SAT2/NIG/PL/JS/KA/1/2017   ........................S......V............G.......................................A...........T.....................................D.NR.V.A.............DYT....P................................A....................  216
*SAT2/NIG/PL/PKN/01/2017    ........................S......V............G.......................................A...........T.....................................D.NR.V.A.............DYT....P................................A....................  216
*SAT2/NIG/PL/PKN/02/2017    ........................S......V............G.......................................A...........T.....................................D.NR.V.A.............DYT....P................................A....................  216
*SAT2/NIG/PL/LANG/02/2017   ........................S......V............G.......................................A...........T.....................................D.NR.V.A........V....DYT....P................................A....................  216
*SAT2/NIG/PL/WAS/01/2017    ........................S......V............G.......................................A...........T.....................................D.NR.V.A.............DYT....P................................A....................  216
*SAT2/NIG/PL/WAS/03/2017    ........................S......V............G.......................................A...........T.....................................D.NR.V.A.............DYT....P................................A....................  216
*SAT2/NIG/BAU/TR/1/2018     ...............................V............G................................V..................T.....................Y...............D.NR.V.A.............GNT..........................T...............................  216
*LAG/IBJ/01/19              .......................G.......V............G...................................................T.R...................................T.NR.V.A..............NT.N.............S.....................A...TD...............  216
*LAG/IBJ/02/19              .......................G.......V............G.....................................................R...................................T.NR.V.A.............SNT.N.............S.....................A...TD...............  216
*KB/005/006/19              ...............................V.................A.......K.T.............S......................T.......................................S.AV.A......V......GNT....P.....Y..V.......................A....................  216
*PL/RY/02/20                .......................G.......V.......................................................Y........T.......................................NRA.AA......V......GNT...................I.....................G................  216
*PL/RY/03/20                .......................G.......V.......................................................Y........T.......................................NRA.AA......V......GNT.........................................GA...............  216
*PL/JS/02/20                .......................G.......V.......................................................Y........T.......................................NRAVAA......V......GNT.........................................GA...............  216
*PL/JS/01/20                .......................G.......V.......................................................Y........T.......................................NRAVAA......V......GNT.........................................GA...............  216
*NIG/PL/157/PLBL/012GF/2020 .......................G.......V.......................................................Y........T.......................................NRA.AA......V......GNT.........................................GA...............  216
*NIG/PL/153/PLBL/008GF/2020 .......................G.......V.......................................................Y........T.......................................NRA.AA......V......GNT....................................S....GA...............  216
*NIG/PL/147/PLBL/002GF/2020 .......................G.......V.......................................................Y........T.......................................NRA.AA......V......GNT.........................................GA...............  216
*NIG/PL/155/PLBL/010GF/2020 .......................G.......V.......................................................Y........T.......................................NRA.AA......V......GNT.........................................GA...............  216
*NIG/PL/151/PLBL/006GF/2020 .......................G.......V.......................................................Y........T.......................................NRA.AA......V......GNT.........................................GA...............  216
*NIG/PL/146/PLBL/001GF/2020 .......................G.......V.......................................................Y........T.......................................NRA.AA......V......GNT.........................................GA...............  216
*NIG/PL/152/PLBL/007GF/2020 .......................G.......V.......................................................Y........T.......................................NRA.AA......V......GNT.........................................GA...............  216
*NIG/PL/149/PLBL/004GF/2020 .......................G.......V.......................................................Y........T.......................................NRA.AA......V......GNT.........................................GA...............  216
*NIG/PL/154/PLBL/009GF/2020 .......................G.......V.......................................................Y........T.......................................NRA..A......V......GNT.........................................GA...............  216
*NIG/PL/148/PLBL/003GF/2020 .......................G.......V.......................................................Y........T.......................................NRA..A......V......GNT.........................................GA...............  216
*NIG/PL/156/PLBL/011GF/2020 .......................G.......V.......................................................Y........T.......................................NRA..A......V......GNT.........................................GA...............  216
*PL/BLD/01/20               .......................G.......V.......................................................Y........T.......................................NRA.AA......V......GNT.........................................GA...............  216
*PL/BLD/07/20               .......................G.......V.......................................................Y........T.......................................NRA.AA......V......GNT.........................................GA...............  216
*PL/BLD/03_25/20            .......................G.......V.......................................................Y........T.......................................NRA.AA......V......GNT.........................................GA...............  216
*PL/BLD/02/20               .......................G.......V.......................................................Y........T.......................................NRA.AA......V......GNT.........................................GA...............  216
*PL/BK/02/20                .......................G.......V.......................................................Y........T.......................................NRA.AA......V......GNT.........................................GA...............  216
*PL/BK/08/20                .......................G.......V.......................................................Y........T.......................................NRA.AA......V......GNT.........................................GA...............  216
*PL/BLD/03_38/20            .......................G.......V.......................................................Y........T.......................................NRA.AA......V......GNT.........................................GA...............  216
*KN/SDW/02/20               .......................G.......V.......................................................Y........T.......................................NRA.AA......V......GNT.........................................GA...............  216
*PL/BK/06/20                .......................G.......V.......................................................Y........T.......................................NRA.AA......V......GNT.........................................GA...............  216
‡NIG/1/14                   ...............................V............G.......................................A...........T.....................................D.NR.V.A.............GNT....P................................A....................  216
‡CHD/7/2016                 ...............................V............G............K......................................T.....................................D.NR.V.A.............DTT....P................................A..................--  214
‡GHA/Tul/2/2018             ...............................V............G...................................................T..H..................................D.NR.V.A.............DNA.A..P.......S........................A....................  216
‡EGY/Sharqiua4/2018         ...............................V............G.........N...........................E...................................................V.K.PVAS.............D.T....P........V.......................A....................  216
‡LIB/40/2012                ...............................V............G.........N..K......................................T..P..................................D.N..V.A.............DNT.............V............................................  216
‡SUD/1/2007                 ...............................V............R.........N.........................................T.........Y...........................N.N....A......T.T....GDT.............V............................................  216
‡NIG/2/2007                 ....................S..........V......................N....T....................................T.....................................N.N....A.............GNT.............V.......................A....................  216
‡Nig/20/12                  ...............................V.................A....N....T....................................T.R.....................................N....A.............GNT..........Y..V.......................A....................  216
‡MAU/2/2014                 ...T...........................V...........................T.............S..............................................................N....AT............G.T..........Y..V.......................A....................  216
‡CAR/911/2012               ......................R........V......................N...........................E.............T.......................................N.A..A.............GNT.............V.........................N................--  214
‡SEN/27/2009                ..............................DV.......A.................K.............................Y................................................N....A......V......GNT.............V.......................A...GD...............  216
‡NIG/1/2012                 ...............................V.................A.........T.............S......................T.......................................N....A.............GNT..........Y..V.......................A....................  216
‡Nig/22/12                  ...............................V.................A....N....T....................................T.R.....................................N....A.............GNT..........Y..V.......................A....................  216
∮GHA/2/90                   ...............T....A.ANP.....DVG...........G..T.E.N............V...A.........T...E.A...........T..........SHNN.......Y...............K.NT.VQ.......V..N...NRK..........Y..........................A...QS...........K.LC  216
∮ZIM/7/83                   ...S................A.T.KK.V..DV..VM..F...L..R.A.A......N..T....L...A...........L.E.E..W........T.T.R......SHNN.....V.Y.........R.....K.TQQS.A......V......NTK.K........Y..........................G....D.....S....KK.LC  216
∮ZIM/14/2002                ....................Q.V.K..M..DV..VM..F.....S..T.N.......D......L.................E.K..Y........T..........SRN..S...L.Y...............K.TDRV.A......V..G...NTR.A........HI...A.....................A...GA...........K.LC  216
∮ETH/1/90                   ......................L.K..M..DV..V...F.....S..T.N.......QH.....L.............T...E.S..Y........T...........HN......V.Y.....V.A.R.....K.TDRVS.......V......D.R.S........H....R.....................A.Q.NN...........K.LC  216
∮BOT/29/98                  ....................S.V.K..M..DV..V...F.....S..T.N....E..D.T....L.................E.K...........T.V........SHNK.......Y...............R.TQKTQA......V..Q...NTR.S........H....EA....................A.E.ND...Y.G.....K.LC  216
∮ETH/2/91                   ............I..T....S.TPA..I..DV...............T.NI................SA.............E.K...........T.T.......YSHN........Y...............D.K.AS.A......V......NTK..........H....A.................A...A...V..............L.  216
∮ETH/2/2007                 ....S........G.A....VANTA..A..DV..........Y......S....E............SA.............E.K...........T.T.........HN........Y...............T.T..S........V.....RDTG.S...................................A.S.N............K.LC  216
∮UGA/19/98                  ...............T....S.RNP..I..DVT................A.................SA.H.............KC..........TV.........SHN........................D.NSKV.A.....QV.QQ...STR.A...................................A...KN.............LY  216
∮UGA/51/75                  ........E......T....S.GAP..V..DV............Q....A...L.............SA......MD.T...E.A...........TNE..E.....SHNK.......Y...............A.S.PVSA.....Q.......SGR.................................A...A....N...........K.LH  216
∮KEN/2/84                   ...............T....T.TAA..V..DV...............T.A.................SA.............E.K...........T..........SHKK.......................K.TEKTIA......V..Q...STK.A...............................A...A.T.QD...........K.L.  216
∮ANG/4/74                   ....................A.TSVK.....V..VM..F........T.EIN....ND.T.I..L..SA...............Q...........T...R.......HNN.....L.Y.R.....A.H.....T.T.KVV.........R....DTK.S...................F....T......A...A....HN..........K.LC  216
∮RWA/1/00                   ...............T....HPNAA.....DI.................A..............V..SA........V......A...........T...........HNK.......Y...............E.T..VSA......V......SGK..................N...............F..A....S...........K.LC  216
```

Figure S3. Amino acid sequence alignment to the SAT2/VII prototype CAR/1/2005 where * represents samples sequenced in this study, ‡ are similar sequence data obtained from genbank and ∮ represents serotype SAT2 prototypes.
